# Characteristics of Korean medical care utilization in patients with traffic injury: Analysis of 3 hospital electronic health record databases

**DOI:** 10.1097/MD.0000000000038495

**Published:** 2024-06-14

**Authors:** Sang-Hyun Lee, Jae-Uk Sul, Jin-Hyun Lee, In Heo, Byung-Cheul Shin, Eui-Hyoung Hwang, Man-Suk Hwang

**Affiliations:** a Department of Korean Medicine, Graduate School, Pusan National University, Yangsan, Gyeongnam, Republic of Korea; b College of Korean Medicine, Dongshin University, Naju, Jeonnam, Republic of Korea; c Department of Korean Rehabilitation Medicine, Dongshin University Korean Medicine Hospital, Gwangju, Republic of Korea; d Institute for Integrative Medicine, Catholic Kwandong University International St. Mary’s Hospital, Incheon, Republic of Korea; e 3rd Division of Clinical Medicine, School of Korean Medicine, Pusan National University, Yangsan, Gyeongnam, Republic of Korea; f Department of Korean Medicine Rehabilitation, Spine and Joint Center, Pusan National University Korean Medicine Hospital, Yangsan, Gyeongnam, Republic of Korea.

**Keywords:** accidents, electronic health records, Korean traditional, medicine, traffic, whiplash injuries

## Abstract

This retrospective study aimed to identify the characteristics of Korean medical care utilization in patients with traffic injury (TI) and to explore the clinical effectiveness of Korean medical interventions for TI through a multicenter chart review. This multicenter, retrospective registry study gathered electronic health records from 3 hospitals between January 1, 2018 and December 31, 2021. Data included treatment dates, demographic information, the Korean Standard Classification of Diseases codes, collision data, Korean medicine treatment modalities, and treatment outcomes. In total, 384 patients (182 inpatients and 202 outpatients) were included in the analysis. Patients were categorized into acute (207 patients, 53.9%), subacute (77 patients, 20.1%), and chronic (100 patients, 26.0%) phases based on the period until the visit. The most frequent Korean Standard Classification of Diseases code was “sprain and strain of cervical spine (S13.4).” All patients, except one, received Korean physiotherapy, followed by acupuncture and cupping. Comparative intragroup analysis revealed significant pain reduction in patients treated with the combination of Chuna manual therapy, herbal medicine, and pharmacopuncture and those treated with pharmacopuncture and herbal medicine only. This study highlights the characteristics of patients with TI visiting medical institutions providing Korean medicine and describes the effectiveness of Korean medicine interventions. Further comprehensive analysis with more data is necessary for future research.

## 1. Introduction

In 2022, Korea recorded 196,836 traffic accidents (TAs) with 2725 fatalities, ranking 4th in deaths per 10,000 cars among OECD countries.^[1]^ Korea dual medical system, encompassing Western and Korean medicine, poses choices for patients involved in TAs.^[2]^ In 2022, 72% of TA patients used Korean medical institutions, including Korean medicine clinics and hospitals, a significant rise from 47.6% in 2018.^[3]^ The reasons for this increased demand are variously attributed to the excellence of Korean medicine, friendlier service, and convergence of Western and Korean medicine.^[4]^ According to survey studies on satisfaction with Korean medicine among patients with TA, acupuncture, pharmacopuncture (PP), and Korean physiotherapy were highly satisfactory.^[5,6]^ Although Korean physiotherapy, PP, and herbal medicine (HM) are not covered by national health insurance,^[7]^ these treatments are covered by the Korean automobile insurance medical fee system, which encourages patients with TA to visit Korean medical institutions when a TA occurs.

While traditional and complementary medicines, including Korean medicine, demonstrate clinical utility, they often face criticism for a lack of comprehensive evidence.^[8]^ Han et al^[9]^ addressed the need for a Korean medicine data registry network to generate evidence on Korean medicine. Therefore, it is imperative to collect and analyze health data held by Korean medical institutions. Given the frequent treatment of traffic injury (TI) in Korean medicine, creating evidence in this area is a top priority.

Accordingly, we recognized the necessity of synthesizing evidence to evaluate the effectiveness, safety, cost-effectiveness, and clinical utility of Korean medicine for TI. Based on the Korean Medicine Clinical Practice Guideline for TI,^[10]^ we aimed to identify the characteristics of Korean medical care utilization of patients with TI and explore the clinical effectiveness of Korean medical interventions through a retrospective chart review. A multicenter, retrospective, exploratory registry study was conducted using electronic health records (EHRs) from 3 hospitals to analyze the characteristics and treatment practices of patients with TI.

## 2. Methods

### 2.1. Study population

We collected EHRs of patients with TI who visited Pusan National University Korean Medicine Hospital (PNUKH), Catholic Kwandong University International St. Mary Hospital (CKUH), and Dongshin University Korean Medicine Hospital (DUKH) from January 1, 2018 to December 31, 2021. The EHR data comprised treatment dates, demographic information, the Korean Standard Classification of Diseases (KCD) codes, collision data, Korean medicine treatment modalities, and treatment outcomes. The study protocol was approved by the institutional review boards of each hospital (PNUKH, 2015001; CKUH, IS21RNMI0093; DUKH, DSGOH_E_2021-010).

The inclusion criteria were: patients, either outpatients or inpatients, who were registered with car insurance from January 1, 2018 to December 31, 2021, at PNUKH, CKUH, and DUKH; and patients who continued to receive treatment for >3 months after the date of the accident. The exclusion criteria were: patients who had not received Korean medicine treatments at least once; and patients with insufficient EHR for analysis (e.g., lack of data such as date of TA or KCD codes).

### 2.2. Data collection

Patient-specific data encompassed treatment dates, including the first date of visit or hospitalization date, date of TA, period from the TA to the first visit (period until the visit), period from the first visit to the most recent visit (treatment period), and date of last treatment or discharge. For hospitalized patients, the readmission status, number of readmissions, and total hospitalization days were also recorded. Patients were classified into the acute, subacute, or chronic phase based on the period from TA to the first visit; we divided patients into the acute phase if it was <21 days, the subacute phase if it was 22 to 90 days, and the chronic phase if it was >91 days. Demographic information included patient sex, age, and past medical history (including surgical history). All principal and additional diagnoses were collected using KCD-8 codes. Collision data contained information regarding the collision type and situation. The collision type included forward, rear-end, side, and multiple pileups. Collision situations were defined as the circumstances when the TA occurred, such as “in-car TA,” “out car TA,” “bicycle TA,” and “motorcycle TA.” Korean medicine treatment modalities included acupuncture, moxibustion, cupping, PP, Chuna manual therapy (CMT), Do-in-conduction exercises, Korean physiotherapy, and HM.^[11–13]^ For hospitalized patients, pain was assessed upon admission and discharge using the numeric rating scale (NRS) to evaluate the effectiveness of Korean medicine treatments. The difference between the NRS at admission and discharge was used to compare the degree of pain reduction in hospitalized patients. NRS is a commonly used pain assessment tool from a scale of 0 (no pain) to 10 (the worst pain imaginable).^[14]^ However, the pain NRS score of outpatients and discharged inpatients were not collected due to a lack of information as some institutions did not record them.

### 2.3. Statistical analysis

Continuous variables which were normally distributed were expressed as mean ± standard deviations (SDs), otherwise as median (Q1, Q3). Categorical data, such as sex, past medical history, and KCD-8 codes, were expressed as number (N) and percentage (%). Intragroup comparisons were tested using the paired t-test or Wilcoxon signed-rank test, while intergroup comparisons were performed with an independent *t* test. Comparisons between 3 groups were conducted using the analysis of variance method. The Pearson chi-squared test was used to analyze categorical data. Statistical significance was determined using a 2-sided test with a significance level of 0.05. For missing data, we used the “pairwise deletion” method.^[15]^

## 3. Results

A total of 384 patients (182 inpatients and 202 outpatients) were included in the analysis. The average patient age was 53.6 years, with 143 (37.2%) male and 241 (62.8%) female patients. The average treatment period for men and women was 32.0 and 33.2 days, respectively. Rear-end collisions were the most common collision type among patients with TI, with “in car TA” as the most common collision situation (Table 1).

**Table 1:** General characteristics of patients with traffic injury.

Variable	Patients			*P* value†
	Overall (n = 384)	Inpatient (n = 182)	Outpatient (n = 202)	
Age (yrs)	53.6 ± 16.6	55.0 ± 16.6	52.3 ± 16.6	.106∫
Sex				
Male	143 (37.2)	64 (35.2)	79 (39.1)	
Treatment period (d)	32.0 ± 34.8	38.1 ± 42.8	27.4 ± 26.5	.073∫
Female	241 (62.8)	118 (64.8)	123 (60.9)	
Treatment period (d)	33.2 ± 35.3	31.5 ± 26.6	34.8 ± 41.6	.486∫
Past history				
Cardiovascular disease	87 (24.0)	50 (28.6)	37 (19.7)	.047∫∫^,^*
Endocrine disease	61 (16.8)	38 (21.7)	23 (12.2)	.016∫∫^,^*
Surgery	96 (26.4)	62 (35.4)	34 (18.1)	.000∫∫^,^*
Collision type				
Rear-end collision	110 (28.6)	58 (31.9)	52 (25.7)	.185∫∫
Forward collision	42 (10.9)	31 (17.0)	11 (5.4)	.000∫∫^,^*
Left side collision	31 (8.1)	17 (9.3)	14 (6.9)	.387∫∫
Right side collision	26 (6.8)	14 (7.7)	12 (5.9)	.495∫∫
Multiple pileup	15 (3.9)	6 (3.3)	9 (4.5)	.558∫∫
Others	81 (21.1)	41 (22.5)	40 (19.8)	.513∫∫
Not applicable	63 (16.4)	38 (20.9)	25 (12.4)	.025∫∫^,^*
Unknown	75 (19.5)	10 (5.5)	65 (32.2)	.000∫∫^,^*
Collision situation				
In car TA	267 (69.5)	124 (68.1)	143 (70.3)	.572∫∫
Out car TA	61 (15.9)	39 (21.4)	22 (10.9)	.005∫∫^,^*
Motorcycle TA	9 (2.3)	8 (4.4)	1 (0.5)	.012∫∫^,^*
Bicycle TA	15 (3.9)	5 (2.7)	10 (5.0)	.266∫∫
Others	32 (8.3)	6 (3.3)	26 (12.9)	.001∫∫^,^*
Intervention				
With CMT	192 (50.0)	96 (52.7)	96 (52.5)	.307∫∫
Without CMT	192 (50.0)	86 (47.3)	106 (47.5)	
With PP	341 (88.8)	167 (91.8)	174 (86.1)	.081∫∫
Without PP	43 (11.2)	15 (8.2)	28 (13.9)	
With HM	250 (65.1)	164 (90.1)	86 (42.6)	.000∫∫^,^*
Without HM	134 (34.9)	18 (9.9)	116 (57.4)	
With CMT, PP, HM	140 (36.5)	88 (48.4)	52 (25.7)	.000∫∫^,^*
With CMT, PP	37 (9.6)	4 (2.2)	33 (16.3)	
With CMT, HM	6 (1.6)	3 (1.6)	3 (1.5)	
With PP, HM	93 (24.2)	65 (35.7)	28 (13.9)	
Only CMT	9 (2.3)	1 (0.5)	8 (4.0)	
Only PP	71 (18.5)	10 (5.5)	61 (30.2)	
Only HM	11 (2.9)	8 (4.4)	3 (1.5)	
None	17 (4.4)	3 (1.6)	14 (6.9)	

All values are presented as mean ± standard deviation or number (%).

∫ Independent *t* test

∫

∫Pearson chi-squared test.

CMT = Chuna manual therapy, HM = herbal medicine, PP = pharmacopuncture, TA = traffic accident.

* *P* < .05.

† *P* value testing the differences between inpatient and outpatient.

Among the 384 patients, 207 were in the acute phase, 77 were in the subacute phase, and 100 were in the chronic phase. Of the 182 inpatients, 162 did not readmit, with the average admission period and period until the visit of 28.3 and 43.5 days, respectively. The mean admission period for acute inpatients was 14.6 days, subacute inpatients 54.5 days, and chronic inpatients 61.2 days, with significant differences between the 3 groups. For the 202 outpatients, the mean treatment period was 31.9 days, and the average period until the visit was 143.0 days. The period until the visit was significantly different for acute, subacute, and chronic patients in both inpatient and outpatient settings (Table 2).

**Table 2:** Analysis of treatment type.

Variables	Patients				*P* value
	Overall	Acute	Subacute	Chronic	
Overall	384 (100)	207 (53.9)	77 (20.1)	100 (26.0)	
Treatment period (d)	32.8 ± 35.1	35.1 ± 24.1	39.4 ± 52.1	22.8 ± 37.9	.004∫^,^*
Inpatient					
Number	182 (100)	124 (68.1)	32 (17.6)	26 (14.3)	
Admission period (d)	28.3 ± 49.4	14.6 ± 14.2	54.5 ± 51.3	61.2 ± 102.0	.000∫^,^*
Number of readmission					
0	162 (89.0)	116 (71.6)	28 (17.3)	18 (11.1)	.001∫∫^,^*
1	12 (6.6)	6 (50.0)	4 (33.3)	2 (16.7)	
2	3 (1.6)	1 (33.3)	–	2 (66.7)	
3	3 (1.6)	1 (33.3)	–	2 (66.7)	
4	1 (0.5)	–	–	1 (100)	
5	1 (0.5)	–	–	1 (100)	
Period until the visit (d)	43.5 ± 90.1	6.0 ± 5.9	45.9 ± 19.3	219.7 ± 137.5	.000∫^,^*
Outpatient					
Number	202 (100)	83 (41.1)	45 (22.3)	74 (36.6)	
Treatment period (d)	31.9 ± 36.6	32.1 ± 21.4	39.4 ± 48.1	27.1 ± 41.5	.209∫
Period until the visit (d)	143.0 ± 380.4	9.3 ± 6.5	50.0 ± 20.3	349.4 ± 574.0	.000∫^,^*

All values are presented as mean ± standard deviation or number (%).

∫ Analysis of variance.

∫

∫Pearson chi-squared test.

* *P* < .05.

A total of 1276 KCD codes were collected, including principal and additional diagnoses. The most frequent KCD code was “sprain and strain of cervical spine (S13.4)” followed by “sprain and strain of lumbar spine (S33.50),” “sprain and strain of shoulder joint (S43.4),” and “concussion, without open intracranial wound (S06.00)” (Table S1, Supplemental Digital Content, http://links.lww.com/MD/M860). For readmitted patients, the most frequent KCD code chapters were “Injury, poisoning and certain other consequences of external causes (S00-T98),” “Diseases of the musculoskeletal system and connective tissue (M00-M99),” and “Diseases of the nervous system (G00-G99).” In “Injury, poisoning and certain other consequences of external causes (S00-T98),” there were 18 cases of fracture, and 11 cases of brain damage (Table S2, Supplemental Digital Content, http://links.lww.com/MD/M861).

All patients, except one, received Korean physiotherapy (383 patients), which was the most frequently utilized Korean medicine intervention. Acupuncture was employed less frequently than Korean physiotherapy in only 1 case (382 patients). Cupping (346 patients), PP (341 patients), and moxibustion (336 patients) were the next most common interventions. Except for Do-in-conduction exercises (16 patients), all interventions were applied to more than half of the patients. Treatment details are outlined in Table S3, Supplemental Digital Content, http://links.lww.com/MD/M862.

Following data collection, there were insufficient information on pain NRS score for 46 cases. Therefore, we classified all of them as missing values and analyzed the pain NRS score for the remaining 136 patients. Further analysis involved examining the treatment effectiveness of Korean medicine by classifying inpatients according to collision type, collision situation, phase, and interventions. All collision types, excluding “unknown,” exhibited a significant decrease in pain for inpatients. In the comparison of treatment effectiveness based on collision situation, “in car TA” and “out car TA” patients showed notable decreases in pain, while “motorcycle TA” and “bicycle TA” patients showed no differences. Notably, all phases showed a significant decrease in the pain NRS score during hospitalization. Evaluation by intervention classifications showed a significant reduction in pain regardless of whether CMT, PP, or HM was performed. When comparing intragroup differences according to combined treatments, patients who received a combination of CMT, HM, and PP and those treated with PP and HM only exhibited significant pain reduction (Table 3).

**Table 3:** Effects of Korean medicine treatment in hospitalized patients.

Variables	No.	Outcome			
		Admission day pain	Discharge day pain	Changes	*P* value
Classification by collision type					
Rear-end collision	51	7.0 (6.0, 8.0)	5.0 (3.0, 6.0)	−1.0 (−3.0, 0.0)	.000∫^,^*
Forward collision	25	7.0 (7.0, 8.0)	5.0 (3.0, 6.0)	−1.0 (−3.0, −1.0)	.000∫^,^*
Left side collision	11	6.82 ± 2.04	4.82 ± 1.99	−2.00 ± 2.05	.009∫∫^,^*
Right side collision	10	6.50 ± 2.12	3.80 ± 2.04	−2.70 ± 2.75	.013∫∫^,^*
Others	27	7.0 (4.0, 7.0)	4.0 (2.0, 7.0)	−1.0 (−2.0, 0.0)	.000∫^,^*
Not applicable	25	7.0 (7.0, 8.0)	5.0 (4.0, 7.0)	−2.0 (−2.0, 0.0)	.000∫^,^*
Classification by collision situation					
In car TA	100	7.0 (5.0, 8.0)	5.0 (3.0, 6.0)	−1.0 (−3.0, 0.0)	.000∫^,^*
Out car TA	27	7.0 (7.0, 8.0)	5.0 (4.0, 7.0)	−2.0 (−2.5, 0.0)	.000∫^,^*
Classification by phase					
Overall	136	7.0 (6.0, 8.0)	5.0 (3.0, 6.0)	−1.0 (−3.0, 0.0)	.000∫^,^*
Acute	91	7.0 (5.0, 8.0)	5.0 (3.0, 6.0)	−1.0 (−2.0, 0.0)	.000∫^,^*
Subacute	28	6.75 ± 1.62	4.43 ± 2.28	−2.32 ± 2.11	.000∫∫^,^*
Chronic	17	8.0 (7.0, 8.0)	7.0 (5.0, 7.0)	−1.0 (−3.0, 0.0)	.003∫^,^*
Classification by CMT					
With CMT	85	7.0 (5.0, 8.0)	5.0 (3.0, 6.0)	−1.0 (−3.0, 0.0)	.000∫^,^*
Without CMT	51	7.0 (6.5, 8.0)	5.0 (3.0, 7.0)	−2.0 (−3.0, 0.0)	.000∫^,^*
Classification by PP					
With PP	128	7.0 (6.0, 8.0)	5.0 (3.0, 6.0)	−1.0 (−3.0, 0.0)	.000∫^,^*
Without PP	8	6.63 ± 1.06	5.13 ± 1.64	−1.50 ± 1.77	.048∫∫^,^*
Classification by HM					
With HM	123	7.0 (6.0, 8.0)	5.0 (3.0, 6.0)	−1.0 (−3.0, 0.0)	.000∫^,^*
Without HM	13	7.0 (7.0, 8.0)	6.0 (4.0, 7.0)	−1.0 (−2.0, 0.0)	.007∫^,^*
Classification by combined treatments					
With CMT, PP, HM	77	7.0 (6.0, 8.0)	5.0 (3.0, 6.0)	−1.0 (−3.0, 0.0)	.000∫^,^*
With PP, HM	41	7.0 (6.0, 8.0)	5.0 (3.0, 7.0)	−2.0 (−3.0, 0.0)	.000∫^,^*

All values are presented as mean ± standard deviation or median (Q1, Q3). Only subgroups with more than 10 patients collected are specified in the table except for the “without PP” subgroup.

∫ Wilcoxon singed-rank test.

∬Paired *t* test.

CMT = Chuna manual therapy, HM = herbal medicine, PP = pharmacopuncture, TA = traffic accident.

* *P* < .05.

## 4. Discussion

This multicenter, retrospective, exploratory registry study investigated the characteristics of Korean medical care utilization in 384 patients with TI who continued to receive treatment for >3 months after the date of the accident. As TA is a psychologically rewarding condition, this study excluded patients who were treated for <3 months, which could have confounded the data, such as early settlement agreements. However, as there was no nontreatment group, the authors tried to be more cautious in determining the clinical utility of Korean medicine and interpreting the results.

The average age of patients was in their 50s, indicating a preference for Korean medicine among older individuals. Interestingly, the highest TA rates were among those in their 20s (18.1%), followed by those in their 30s (17.7%),^[1]^ but the utilization of Korean medicine was uncommon. This may be due to the lack of awareness of Korean medicine among young people,^[16]^ and treatment periods < 3 months. Considering that TI has long sequelae, strategies to encourage young adults to utilize Korean medicine for TI are crucial.^[17]^ Furthermore, sex disparities were noted in 2022, with men and women accounting for 62.4% and 37.6% of TA injuries, respectively.^[1]^ However, in this study, women accounted for 62.8%, which is 1.7 times higher than that of men. This implies that men with TI are not actively treated for reasons such as early return to work,^[18]^ emphasizing the need for implementing policies for equitable sex-based Korean medicine utilization.

The number of people injured in rear-end collisions was 55,193, with 98,331 injured in side collisions in 2022.^[1]^ However, rear-end collisions were the predominant collision type in this study. Under current Korean law, it is difficult to determine 100% of faults in side collisions, while 100% of faults are available in rear-end collisions, making rear-end collision victims more active in treatment.^[19]^

TA causes immediate clinical symptoms, such as pain or limited range of motion, because it is caused by direct trauma from vehicle collisions.^[20]^ According to the Korean automobile insurance medical fee system, the coverage of car insurance decreases with time from the date of the accident. The Korean Health Insurance Review & Assessment Service limits the coverage of patients with TI to hospitalization within 3 days from the date of the accident and only allows 5 days of hospitalization. Accordingly, patients tend to visit hospitals during the acute phase. Notably, 11% of inpatients were readmitted more than once, indicating persistent symptoms even after discharge; 60% of the readmitted patients were in the subacute or chronic phase, indicating that the longer symptoms persisted, the more repeated hospitalizations were required. In addition, patients with fractures or brain damage tended to be readmitted frequently, indicating the need for readmission or longer treatment for severe injuries. The proportion of outpatients in the acute phase was lower, while the proportion of patients in the subacute and chronic phases was higher than that of inpatients. Data indicate that patients in the acute phase tend to be hospitalized actively, and these patients switch to outpatient treatment after discharge, which results in a higher proportion of outpatients in the subacute and chronic phases.

Due to the mechanism of TA, sprains and strains were the most common KCD codes among patients with TI. Besides musculoskeletal codes, other codes related to systemic symptoms, such as dizziness (R42) and headache (G44.3), were also common. In severe cases, intervertebral disc displacement (M51.2) occurred because of TAs. Inpatients had a higher ratio of codes related to concussions (S06.00) than outpatients, suggesting that patients with head trauma were more likely to require hospitalization. Symptoms related to head trauma such as headache, dizziness, and memory impairment make social activities difficult,^[21]^ resulting in patients with head trauma preferring hospitalization because of psychological concerns about the occurrence of traumatic brain injury.^[22]^

In the analysis of Korean medicine interventions, HM, whose prescription is limited to a maximum of 7 days per accident, was performed in 90% of inpatients and only 43% of outpatients. As stated earlier, this was presumed to be due to the transition from inpatient to outpatient treatment after discharge. In addition, only half of the patients received CMT, which was lower than that of the other interventions. Because most patients were in the acute phase, early application of CMT may worsen the symptoms; therefore, Korean medicine doctors screen patients carefully before proceeding with CMT. Treatment effectiveness was noteworthy among patients receiving combined therapies, specifically CMT, HM, and PP together, emphasizing their potential benefits. However, as the treatments were not performed in a controlled environment, the efficacy of other combined therapies needs to be verified in future studies.

The significance of this study lies in revealing patient characteristics and describing the effectiveness of Korean medicine treatments using the NRS. Compared to studies that retrospectively analyzed the EHR of patients with TI by the Automobile Accident Compensation Securities Law, which has been revised 14 times in the past 10 years, this study has strengths in the following aspects.^[23–26]^ First, the effectiveness of the interventions was described. As this study aimed to synthesize evidence to explore the effectiveness of Korean medicine interventions, the effectiveness of each combination of CMT, PP, and HM was documented. Second, a multicenter study was conducted. Previous studies were conducted at a single Korean medicine clinic or hospital and only reflected regional demographic characteristics. In this study, we collected data from 3 hospitals, making it possible to establish a common registry and secure more objective and extensive data. Third, we compared inpatients and outpatients separately. Previous studies analyzed only 1 group of outpatients or inpatients. The differences between the groups were analyzed in this study.

However, this study has some limitations. First, as the sample size was small, the results cannot represent the whole patients with TI and only show partial trends. In addition, the small sample size made it difficult to stratify the analysis by centers, providing a potential confounding factor that might affect the study results. Second, an accurate analysis could not be performed because of incomplete information. As this study had a retrospective, single-group non-interventional design, it was difficult to collect accurate information, which was not suitable for between-group analysis. Third, the characteristics of traffic related injuries presented in this study are mostly body parts injured. A number of important factors related to TI, such as 30-day mortality or use of safety equipment, are not covered. In the future, it will be necessary to analyze the characteristics of Korean medical care utilization in patients with TI with sufficient information to demonstrate the effectiveness and safety of Korean medicine and to establish a Korean medicine data registry network.

## 5. Conclusions

As there is a lack of common data registry network in Korean medicine, it was necessary to analyze the actual utilization of Korean medicine in patients with TI to generate evidence on Korean medicine. Accordingly, we analyzed the data of patients with TI at 3 hospitals through a retrospective chart review. As a result, patients in their 50s were the most common age group, and female patients outnumbered male ones. Inpatients were more likely to be in the acute phase, while outpatients were more likely to be in the subacute and chronic phases. Regardless of the collision type, collision situation, phase, and Korean medicine interventions, inpatients had significant pain reduction while hospitalization. This study has its meaning in providing valuable insights into the management of patients with TI through Korean medicine and potentially aiding healthcare practitioners in their approach to treating patients with TI.

## Acknowledgments

We would like to thank Editage (www.editage.co.kr) for English language editing.

## Author contributions

**Conceptualization:** Sang-Hyun Lee, Man-Suk Hwang.

**Funding acquisition:** Sang-Hyun Lee.

**Writing – original draft:** Sang-Hyun Lee.

**Writing – review & editing:** Sang-Hyun Lee, Man-Suk Hwang.

**Investigation:** Jae-Uk Sul, Jin-Hyun Lee, In Heo, Byung-Cheul Shin, Eui-Hyoung Hwang, Man-Suk Hwang.

**Methodology:** In Heo, Byung-Cheul Shin.

**Supervision:** In Heo, Byung-Cheul Shin, Eui-Hyoung Hwang, Man-Suk Hwang.

**Project administration:** Man-Suk Hwang.

## Supplementary Material






