# Supplementary material for: Characteristics of Korean medical care utilization in patients with traffic injury: Analysis of 3 hospital electronic health record databases
Source: Medicine (Baltimore). 2024 Jun 14;103(24):e38495. doi: 10.1097/MD.0000000000038495 (PMC11175872; doi:10.1097/MD.0000000000038495)
Supplement: Supplementary file 1 [file medi-103-e38495-s001.docx]

| **Table S1. Distribution by KCD-8 Code of Patients** | | | |
| --- | --- | --- | --- |
| Ranking | Classification | Number | Percent |
| Overall |  | 1,276 | 100 |
| 1 | Sprain and strain of cervical spine(S13.4) | 242 | 19.0 |
| 2 | Sprain and strain of lumbar spine(S33.50) | 227 | 17.8 |
| 3 | Sprain and strain of shoulder joint(S43.4) | 78 | 6.1 |
| 4 | Concussion, without open intracranial wound(S06.00) | 48 | 3.8 |
| 5 | Sprain and strain of other and unspecified parts of knee(S83.6) | 27 | 2.1 |
| 6 | Concussion of knee(S80.0) | 21 | 1.6 |
| 7 | Headache(R51) | 17 | 1.3 |
| 8 | Multiple superficial injuries, unspecified(T00.9) | 15 | 1.2 |
| 9 | Dizziness and giddiness(R42)  Sprain and strain of thoracic spine(S23.3)  Sprain and strain of wirst, part unspecified(S63.59) | 13 | 1.0 |
| 12 | Chronic post-traumatic headache(G44.3) | 11 | 0.9 |
| 13 | Other specified intervertebral disc displacement(M51.2) | 10 | 0.8 |
| Inpatient |  | 672 | 100 |
| 1 | Sprain and strain of lumbar spine(S33.50) | 103 | 15.3 |
| 2 | Sprain and strain of cervical spine(S13.4) | 102 | 15.2 |
| 3 | Concussion, without open intracranial wound(S06.00)  Sprain and strain of shoulder joint(S43.4) | 36 | 5.4 |
| 5 | Sprain and strain of other and unspecified parts of knee(S83.6) | 14 | 2.1 |
| 6 | Concussion of knee(S80.0) | 11 | 1.6 |
| 7 | Multiple superficial injuries, unspecified(T00.9) | 10 | 1.5 |
| Outpatient |  | 604 | 100 |
| 1 | Sprain and strain of cervical spine(S13.4) | 140 | 23.2 |
| 2 | Sprain and strain of lumbar spine(S33.50) | 124 | 20.5 |
| 3 | Sprain and strain of shoulder joint(S43.4) | 42 | 7.0 |
| 4 | Sprain and strain of other and unspecified parts of knee(S83.6) | 13 | 2.2 |
| 5 | Concussion, without open intracranial wound(S06.00) | 12 | 2.0 |
| 6 | Headache(R51) | 11 | 1.8 |
| 7 | Sprain and strain of thoracic spine(S23.3)  Concussion of knee(S80.0) | 10 | 1.7 |
| Only diseases with more than 10 patients collected are specified in the table. | | | |
