# Supplementary material for: Characteristics of Korean medical care utilization in patients with traffic injury: Analysis of 3 hospital electronic health record databases
Source: Medicine (Baltimore). 2024 Jun 14;103(24):e38495. doi: 10.1097/MD.0000000000038495 (PMC11175872; doi:10.1097/MD.0000000000038495)
Supplement: Supplementary file 2 [file medi-103-e38495-s002.docx]

| **Table S2. Distribution by KCD-8 Code Chapter in Readmitted Patients** | | | |
| --- | --- | --- | --- |
| Ranking | Classification | | Number |
| 1 | Injury, poisoning and certain other consequences of external causes (S00-T98) | | 54 |
|  |  | Codes for fracture | 18 |
|  |  | Codes for brain damage | 11 |
| 2 | Diseases of the musculoskeletal system and connective tissue (M00-M99) | | 6 |
| 3 | Diseases of the nervous system (G00-G99) | | 5 |
| 4 | Diseases of the eye and adnexa (H00-H59) | | 3 |
| 4 | Codes for special purposes (U00-U99) | | 3 |
| 6 | Mental and behavioural disorders (F00-F99) | | 2 |
| 6 | Diseases of the circulatory system (I00-I99) | | 2 |
| 8 | Certain infectious and parasitic diseases (A00-B99) | | 1 |
| 8 | Diseases of the skin and subcutaneous tissue (L00-L99) | | 1 |
| 8 | Symptoms, signs and abnormal clinical and laboratory findings (R00-R99) | | 1 |
|  | | | |
