# Supplementary material for: Characteristics of Korean medical care utilization in patients with traffic injury: Analysis of 3 hospital electronic health record databases
Source: Medicine (Baltimore). 2024 Jun 14;103(24):e38495. doi: 10.1097/MD.0000000000038495 (PMC11175872; doi:10.1097/MD.0000000000038495)
Supplement: Supplementary file 3 [file medi-103-e38495-s003.docx]

| **Table S3.** **Analysis of Korean Medicine Treatment Modalities** | | | |
| --- | --- | --- | --- |
|  | Intervention | Number | Percent |
| Overall |  | 384 | 100 |
| 1 | Korean physiotherapy | 383 | 17.1 |
| 2 | Acupuncture | 382 | 17.0 |
| 3 | Cupping | 346 | 15.4 |
| 4 | Pharmacopuncture | 341 | 15.2 |
| 5 | Moxibustion | 336 | 15.0 |
| 6 | Herbal medicine | 250 | 11.1 |
| 7 | Chuna manual therapy | 192 | 8.5 |
| 8 | Do-in-conduction exercises | 16 | 0.7 |
| Inpatient |  | 182 | 100 |
| 1 | Acupuncture  Korean physiotherapy | 182 | 15.9 |
| 3 | Moxibustion | 174 | 15.2 |
| 4 | Cupping | 173 | 15.1 |
| 5 | Pharmacopuncture | 167 | 14.6 |
| 6 | Herbal medicine | 164 | 14.3 |
| 7 | Chuna manual therapy | 96 | 8.4 |
| 8 | Do-in-conduction exercises | 9 | 0.8 |
| Outpatient |  | 202 | 100 |
| 1 | Korean physiotherapy | 201 | 18.3 |
| 2 | Acupuncture | 200 | 18.2 |
| 3 | Pharmacopuncture | 174 | 15.8 |
| 4 | Cupping | 173 | 15.7 |
| 5 | Moxibustion | 162 | 14.7 |
| 6 | Chuna manual therapy | 96 | 8.7 |
| 7 | Herbal medicine | 86 | 7.8 |
| 8 | Do-in-conduction exercises | 7 | 0.6 |
|  | | | |
